# Two remarkable new species of *Prothemus* Champion from China and Thailand, with comments on their systematic status (Coleoptera, Cantharidae)

**DOI:** 10.3897/zookeys.119.1621

**Published:** 2011-07-15

**Authors:** Yuxia Yang, Sergey V. Kazantsev, Xingke Yang

**Affiliations:** 1 College of Life Science, Hebei University, Baoding 071002, Hebei Province, China; 2 Insect Centre, Doneskaya str., 13-326, Moscow 109651, Russia; 3 Key Laboratory of Zoological Systematics and Evolution, Institute of Zoology, Chinese Academy of Sciences, Beijing 100101, China

**Keywords:** Coleoptera, Cantharidae, *Prothemus*, new species, China, Thailand

## Abstract

Two remarkable new cantharid species were described, *Prothemus laticornis* **sp. n.** (CHINA: Yunnan) and *Prothemus lycoceroides* **sp. n.** (THAILAND: Chiang Dao), and provided with illustrations of aedeagi, ultimate abdominal sternites and antennae of both sexes. Their systematic status was discussed, because they differed from all other species of *Prothemus* in the dilated and/or serrate antennae and elytra with distinct longitudinal costae and longer pubescence.

## Introduction

The genus *Prothemus* was proposed by Champion in 1926, with *Prothemus neglectus* originally designated as the type species. Most of the taxonomic work on this genus was contributed by Wittmer (1954a,b, 1972, 1973, 1981, 1982, 1984, 1987, 1993, 1995, 1997a,b), besides, (Okushima and Satô (1997, 2002), Okushima (2008) and (Švihla (2004, 2005, 2011) added some species. Now this genus has 62 species (including the new species described here), distributed in the Oriental (20 species) and East Palaearctic (42 species) regions (Kazantsev and Brancucci 2007).

Recently, we received two interesting cantharid species from China and Thailand respectively. By the external characters, it was not easy to determine whether they belonged to *Prothemus* or *Lycocerus* Gorham, 1889,or a new genus, because their tarsal claws (pro- and meso-outer tarsal claws each roundly appendiculate in male, while simple in female) and rounded pronotum matched the diagnosis of *Prothemus*, while their serrate antennae and pubescent elytra, which characters were common in some *Lycocerus* species, have not been reported in *Prothemus*. After studying the aedeagi of the two species, we discovered that they were of the typical *Prothemus* type. Based on all the above characters, also taking into account that the same variation in the antennae and pubescence occurred in other genera of Cantharinae, such as *Fissocantharis* Pic, 1921, *Lycocerus* Gorham, 1889 and *Habronychus* Wittmer, 1981, we considered that these species to be the members of *Prothemus*, and described them here under the names of *Prothemus laticornis* sp. n. and *Prothemus lycoceroides* sp. n..

## Material and methods

The type specimens are deposited in the Institute of Zoology, Chinese Academy of Sciences, Beijing, China (IZAS), Hebei University Museum, Baoding, China (HBUM) and Dr. SV Kazantsev’s private collection at Insect Centre, Moscow, Russia (ICCM).

The aedeagi were detached from the body under a stereoscopic microscope and kept in 10% KOH solution for several minutes, then cleared in 75% alcohol and observed under a compound light microscope. Line figures were drawn with the aid of a camera lucida mounted on a Nikon SMZ 800 stereomicroscope. The scanning electronic micrographs were edited in CORELDRAW 12 and ADOBE PHOTOSHOP 8.0.1. The habitus photos were taken by Canon 450D digital camera with a Canon EF 100mm f/2.8 USM Macro Lens. The body length was measured from the anterior margin of clypeus to apex of elytron, and width was at the point of maximum width of the conjoint elytra. Absolute measurements were used in millimetres (mm).

## Taxonomy

### Prothemus laticornis

Y. Yang & X. Yang sp. n.

urn:lsid:zoobank.org:act:445C8C99-D76C-4A9F-8CBA-07B2143F15D8

http://species-id.net/wiki/Prothemus_laticornis

Figs 1 3–9 

#### Type material.

 **Holotype** male, CHINA: Yunnan, W. Cangshan, 2100m, 3.vi.2008 (IZAS). Paratypes: one male, Yunnan, Cangshan, 21.v.2009, leg. Shoubin Li (HBUM); one female, Yunnan, Yangbi, Xueshanhe, 2000–2800m, 6–8.vi.2004, leg. Song Wang (IZAS); one female, same data as the latter, leg. Xueyan Guo (IZAS); one female, Yunnan, E. slope Cangshan, Mojian, 2.vi.2009, leg. Jianhui Zeng (HBUM)[above transferred from Chinese labels]; two females, Yunnan, Gaoligong Mts., 1500–2500m, 25.22°N, 98.49°E, 17–24.v.1995, leg. V. Kubáň (ICCM).

#### Distribution.

 China (Yunnan).

#### Diagnosis.

This new species can be easily distinguished from all others of *Prothemus* by the serrate antennae and elytra with distinct longitudinal costae and longer pubescence.

#### Description.

**Figures 1–2.**
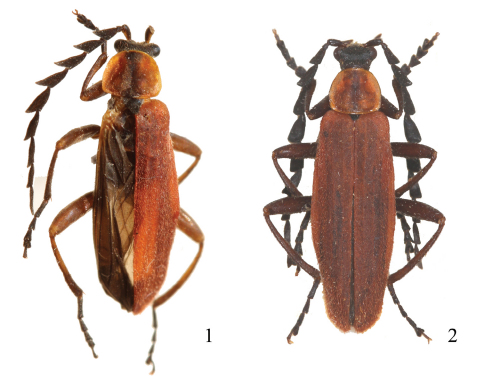
Male habitus, dorsal view **1** *Prothemus laticornis* sp. n. **2** *Prothemus lycoceroides* sp. n.

 **Male** (Fig. 1). Black. Pronotum and elytra red; pronotum with a darkened median longitudinal stripe. Body surface densely covered with black pubescence, except on pronotum and elytra with reddish brown pubescence, slightly longer on elytra.

**Figures 3–9.**
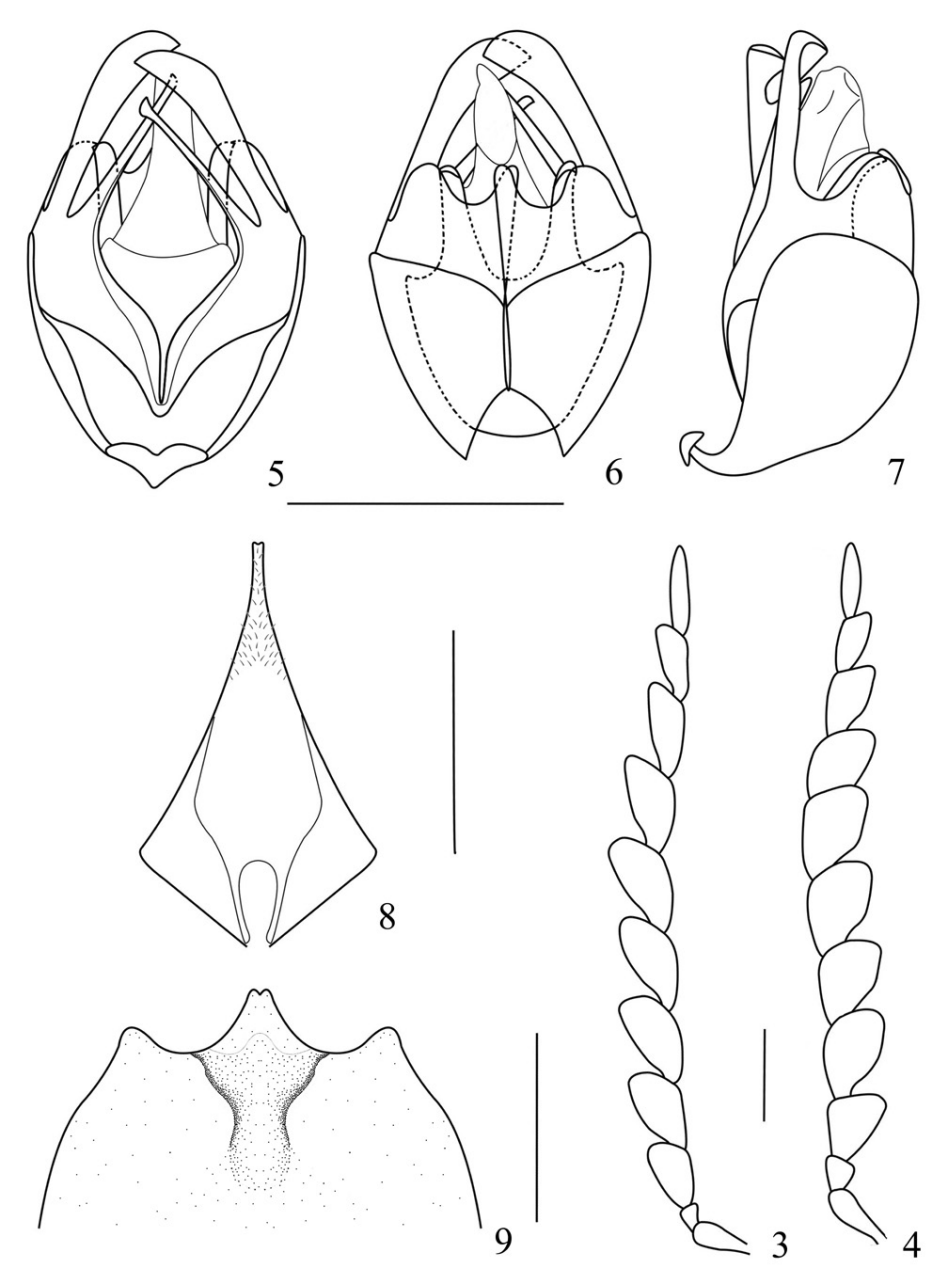
*Prothemus laticornis* sp. n. **3** male left antenna, dorsal view **4** female left antenna, dorsal view **5–7** aedeagus (**5** ventral view **6** dorsal view **7** lateral view) **8** male abdominal sternite IX, ventral view **9** female abdominal sternite VIII, ventral view. Scale bars: 1 mm.

Head surface densely and finely punctate, slightly depressed on vertex; eye slightly protruding, breadth across eyes narrower than maximum width of pronotum; ultimate maxillary palpomere elongate, securiform, widest near base, acute at apex; antenna (Fig. 3) extending to apical one-third of elytron, antennomere II as long as wide at apex, III–X flattened and subserrate, widened apically, protuberant and rounded at apical inner angles, no more than 1.5 times as long as wide at apices, XI flattened and subparallel-sided, pointed at apex.

Pronotum slightly wider than long, widest near base, anterior and lateral margins rounded, posterior margins nearly straight, all angles rounded, disc distinctly convex, with an obscure median longitudinal groove.

Elytra about 5 times longer than pronotum and 5 times longer than wide at humeri, humeral width distinctly wider than that of posterior margin of pronotum, outer margins distinctly diverging posteriorly and slightly converging at apical one-third, disc surface densely and very finely punctuated, with 3 distinct longitudinal costae.

Abdominal sternite IX (Fig. 8) strongly narrowed apically, and far from extending to lateral margins of ventrite IX in natural state.

Aedeagus (Figs 5–7) : dorsal plate of each paramere with lateral trunk entirely located in ventral side and slightly widened near apex, middle plate distinctly protuberant and roundly emarginated near inner angle, ventral process of each paramere slightly widened and acute at inner angle, laterophysis stout and as long as middle plate of dorsal plate of each paramere.

**Female**. Body larger and wider, eye less protruding, antenna (Fig. 4) shorter and wider, pronotum wider than that of male, abdominal sternite VIII (Fig. 9) with a large deep impression and membranous protuberance in middle of posterior margin.

**Variation in type series.** Legs sometimes mostly brown, pronotum sometimes entirely reddish brown, or with two black markings near middle of anterior and posterior margins respectively. Body length 9.0–13.0 mm, width 3.0–5.0 mm.

#### Etymology.

 This new specific name is derived from Latin words *latus* (wide) and *cornu* (antenna), referring to its dilated antennae.

#### Remarks.

The holotype was damaged, its left elytron and right antennomeres VIII-XI were missing. Paratypes: the male with left antennomeres III–XI and right IX–XI and left pro-leg, one female with left antennomeres III–XI and another female with right antennomeres III–XI were missing.

### Prothemus lycoceroides

Kazantsev sp. n.

urn:lsid:zoobank.org:act:3E254644-AAE8-473B-AB6A-FF3163B4A4EF

http://species-id.net/wiki/Prothemus_lycoceroides

Figs 2 10–12 

#### Type material.

 **Holotype** male, THAILAND, Chiang Dao, 1000m, 19°25'N, 98°52'E, 17–24.v.1991, leg. V. Kubáň (ICCM). Paratypes, two males and one female, same data as holotype (ICCM).

#### Distribution.

Thailand (Chiang Dao).

#### Diagnosis.

*Prothemus lycoceroides* sp. n. is fairly similar to *Prothemus laticornis* sp. n., but distinguishable by the narrower antennae, less prominent longitudinal elytral costae and less curved apices of parameres of the aedeagus.

#### Description.

 **Male** (Fig. 2). Black. Pronotum and elytra red; pronotum with a darkened median stripe.

**Figures 10–12.**
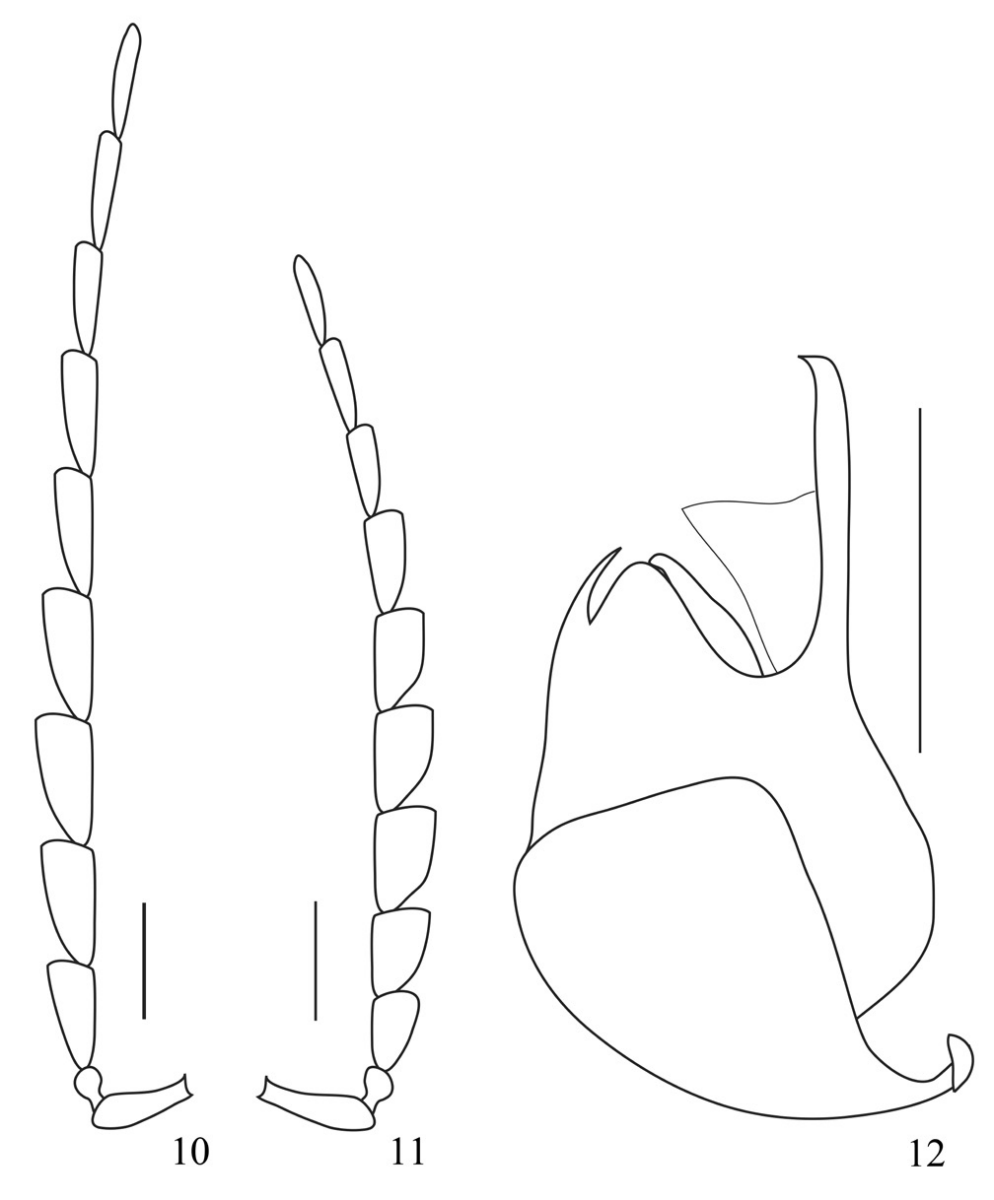
*Prothemus lycoceroides* sp. n. **10** male right antenna, dorsal view **11** female left antenna, dorsal view **12** aedeagus (lateral view). Scale bars: 1 mm.

Head surface dorsally densely and finely punctate, slightly depressed between eyes; ultimate maxillary palpomere elongate, securiform, widest near base, acute at apex; antenna extending to apical one-fourth of elytron, antennomere II as long as wide at apex, III–VIII flattened and serrate, at least 2.0 times as long as wide at apices, IX–XI slightly flattened and subparallel-sided (Fig. 10).

Pronotum slightly wider than long, widest near base, all margins and angles rounded, disc distinctly convex, with an obscure median groove.

Elytra long, about 4.75 times longer than pronotum and 3.2 times longer than wide at humeri, humeral width distinctly wider than that of posterior margin of pronotum, almost parallel-sided, disc surface densely and finely punctate, with 2 distinct longitudinal costae.

Aedeagus (Fig. 12): fairly similar to *Prothemus laticornis* sp. n., distinguishable by less curved apices of parameres.

**Female**. Similar to male, but larger and wider, eye smaller, antenna (Fig. 11) shorter and wider in middle antennomeres.

Body length 13.2–13.8 mm, width 3.0–3.3 mm.

#### Etymology.

The name of the new species is modified from *Lycocerus*, alluding to its similarity to the members of some *Lycocerus* species.

#### A key to distinguish the new species from all other species of Prothemus Champion

**Table:** 

1	Antennae dilated and or serrate; elytra covered with distinct longitudinal costae and longer pubescence	2
–	Antennae filiform; elytra never like above	all other species
2	Antennae wider, male antennomeres III–VIII no more than 1.5 times as long as wide at apices, IX–X distinctly widened	*Prothemus laticornis* sp. n.
–	Antennae narrower, male antennomeres III–VIII at least 2.0 times as long as wide at apices, IX–X slightly widened	*Prothemus lycoceroides* sp. n.

## Discussion

We were greatly surprised to see these strange species at first. Most of their morphological characters showed that they undoubtedly had to be placed in the subfamily Cantharinae. However, judging from the appearance, it was difficult for us to refer them to any genus. Their tarsal claws and pronotum matched the diagnosis of *Prothemus* very well, but they had dilated and/or serrate antennae and elytra with distinct longitudinal costae and slightly longer reddish brown pubescence, which were hitherto unknown in *Prothemus*, although common in some *Lycocerus* species. Dissection of their male copulatory organs showed they had the typical aedeagi of *Prothemus*. Based on external characters and male genital structures, we supposed that these species should be attributed to *Prothemus*, and their character states of elytra and antennae were considered to be apomorphic within the genus *Prothemus*. In the subfamily Cantharinae, the same variations in antennae and elytra also occurred in the genera *Fissocantharis*, *Habronychus* and *Lycocerus*. Hypothetically they mimic one another and/or some other beetle groups, such as Lycidae, in order to benefit from having similar appearance, for example, to protect themselves better or find more effective ways of attacking prey.

This study provided further proof that appearance of soldier-beetles within a single genus, at least in the subfamily Cantharinae, can vary significantly, while the aedeagal structures appear to be more conservative, apparently because they are subject to the selection pressure of the environment to a lesser extent than most external characters.

## Supplementary Material

XML Treatment for Prothemus laticornis

XML Treatment for Prothemus lycoceroides
